# Color Doppler Imaging Assessment of Ocular Blood Flow Following Ab Externo Canaloplasty in Primary Open-Angle Glaucoma

**DOI:** 10.3390/jcm13237373

**Published:** 2024-12-03

**Authors:** Mateusz Zarzecki, Jakub Błażowski, Iwona Obuchowska, Andrzej Ustymowicz, Paweł Kraśnicki, Joanna Konopińska

**Affiliations:** 1 Department of Ophthalmology, Medical University of Bialystok, 15-089 Bialystok, Poland; blazowski.jakub@gmail.com (J.B.); iwona.obuchowska@umb.edu.pl (I.O.); pawel.krasnicki@umb.edu.pl (P.K.); joanna.konopinska@umb.edu.pl (J.K.); 2 Department of Radiology, Medical University of Bialystok, 15-089 Bialystok, Poland; andrzej.ustymowicz@umb.edu.pl

**Keywords:** glaucoma, primary open-angle glaucoma, canaloplasty, color Doppler imaging, ocular blood flow

## Abstract

**Background/Objectives**: Glaucomatous neuropathy, a progressive deterioration of retinal ganglion cells, is the leading cause of irreversible blindness worldwide. While elevated intraocular pressure (IOP) is a well-established modifiable risk factor, increasing attention is being directed towards IOP-independent factors, such as vascular alterations. Color Doppler imaging (CDI) is a prominent technique for investigating blood flow parameters in extraocular vessels. This prospective, nonrandomized clinical trial aimed to assess the impact of ab externo canaloplasty on ocular blood flow parameters in patients with primary open-angle glaucoma (POAG) at a three-month follow-up. **Methods**: Twenty-five eyes of twenty-five patients with early or moderate POAG underwent canaloplasty with simultaneous cataract removal. CDI was used to measure peak systolic velocity (PSV), end-diastolic velocity (EDV), and resistive index (RI) in the ophthalmic artery (OA), central retinal artery (CRA), and short posterior ciliary arteries (SPCAs) before and after surgery. **Results**: The results showed a significant reduction in IOP and improvement in mean deviation at three months post-surgery. Best corrected visual acuity and retinal nerve fiber layer thickness significantly increased at each postoperative control visit. However, no significant changes were observed in PSV, EDV, and RI in the studied vessels. **Conclusions**: In conclusion, while canaloplasty effectively reduced IOP and medication burden, it did not significantly improve blood flow parameters in vessels supplying the optic nerve at three months post-surgery. Careful patient selection considering glaucoma severity and vascular risk factors is crucial when choosing between canaloplasty and more invasive procedures like trabeculectomy. Further larger studies are needed to comprehensively analyze this issue.

## 1. Introduction

Glaucomatous neuropathy, defined as the progressive deterioration of retinal ganglion cells, is the leading cause of irreversible blindness worldwide. Elevated intraocular pressure (IOP) is the only well-studied, established modifiable risk factor for its development [1]. However, an increasing number of scientists are directing their attention to the considerable population of patients who develop glaucomatous neuropathy despite effective IOP control [2,3]. This has led a significant number of researchers to investigate new IOP-independent risk factors for the development and progression of the condition. Among these, vascular factors are among the most well documented in the literature. Any alteration of the vascular bed involving a decrease in perfusion pressure and an increase in resistance index in the ocular vessels that exceeds the capacity of autoregulatory mechanisms results in a reduction in blood flow in the vasculature that supplies the optic nerve. This, in turn, leads to ischemia and subsequent damage to the retinal ganglion cell fibers [4]. The ultimate consequence of these pathological processes is the progression of glaucomatous neuropathy [5].

The degree of response to perfusion disturbances in the extraocular circulation varies considerably between individuals, a phenomenon that is characteristic of autoregulatory mechanisms [6]. The contractility of the smooth muscle cells involved in autoregulatory processes is determined by endothelial cells through the release of vasoactive factors with opposing effects, including nitric oxide (NO), which has a vasodilatory effect, and endothelin-1 (ET-1), characterized by strong vasoconstrictive properties [7,8,9]. Several published studies have highlighted the elevation of ET-1 levels in serum and aqueous humor in patients with glaucoma, which may be related to progressive neuropathy of the optic nerve caused by impaired perfusion [10,11]. Similarly, well-recognized circulatory risk factors such as hypertension, hypotonia, vasoconstrictive syndromes (migraine, Raynaud’s disease, Flammer syndrome), diabetes, hypercholesterolemia, or blood clotting disorders may contribute to damage to the endothelium of blood vessels supplying the globe [12,13,14,15,16,17,18,19]. However, other studies indicate that glaucoma is a direct cause of the disruption of autoregulatory mechanisms [5,20,21,22]. The question of whether glaucoma or concomitant risk factors are the primary pathology responsible for impaired perfusion of the ocular circulation remains unresolved [23,24].

Ab externo canaloplasty is a modern and safe surgical technique used in the surgical treatment of open-angle glaucoma (POAG) [25]. The advantages of this technique include the absence of a filtering bleb, which eliminates the need for antimetabolites, a lower complication rate, and fewer additional procedures in the postoperative period [26]. Canaloplasty is highly effective in permanently lowering IOP, although it has a less pronounced IOP-lowering potential than trabeculectomy, which is still considered the gold standard [27,28]. As a nonperforating procedure, it involves increasing the wall tension of Schlemm’s canal (SC) over a 360-degree area of its circumference. Its IOP-lowering action is based on three mechanisms: viscodilatation of the SC, increase in TM tension, and percolation of aqueous humor (AH) through the trabecular–Descemet window (TDW) into the intrascleral space. Due to the tight suturing of the superficial scleral flap, AH does not enter the subconjunctival space but remains in the intraocular lake, from where it can diffuse into the suprachoroidal space and drain out of the eyeball through the suprachoroidal route [29]. The average diameter of the catheter is 250 μm, while the diameter of the SC is 150–350 µm. Histological results have shown that SC cannulation with a catheter followed by the placement of a tension suture compresses the outer wall and disrupts the endothelial adhesions between the TM and the entrance to the collector channels, thus eliminating the main resistance point of OUT for AH outflow. AH flows through the ostia of the collectors into the aqueous veins and from there into the systemic circulation. There are also reports of a hypothetical role for viscoelastic as an antifibrotic and anti-inflammatory agent [30]. However, histological findings are inconclusive [31].

One of the most prominent and widely utilized innovative techniques in clinical practice for the investigation of blood flow parameters in extraocular vessels is color Doppler imaging (CDI) [32]. The measurement of peak systolic velocity (PSV) and end-diastolic velocity (EDV) enables the determination of the resistive index (RI), which is elevated in cases of hemostatic disorders of the ocular circulation. The safety, non-invasiveness, high availability, and cost-effectiveness of this technique have resulted in its widespread use in research methodology [33,34]. It has been observed by numerous authors that abnormal Doppler blood flow parameters are prevalent in the ocular artery (OA), central retinal artery (CRA), and posterior short ciliary arteries (SPCAs) in patients with POAG when compared to the healthy population [32,33,34,35,36,37,38,39]. Furthermore, a correlation was identified between the severity of glaucomatous neuropathy and the magnitude of hemodynamic disturbances in the orbital circulation [40]. The researchers indicate the potential utility of CDI in the detection and monitoring of glaucoma as a methodology for the assessment of ocular circulation and hemostasis abnormalities, which could facilitate the implementation of a more comprehensive and individualized therapeutic approach for the condition [34].

The most recent recommendations from both European and global glaucoma societies advise limiting the treatment of this disease to topical drops in glaucoma, taking into account the impact on patient quality of life and the minimization of side effects [41].

In light of the evidence that the root cause of glaucoma may be a disruption of the blood supply to the optic nerve, the objective of this study was to ascertain whether a nonpenetrating surgical method of treating glaucoma, such as ab externo canaloplasty, has an impact on blood flow in the ocular vascular bed. There is a paucity of literature on the effects of glaucoma surgery on the hemodynamics of the extraocular circulation. Most of these studies focus on traditional bleb-dependent surgical techniques, such as trabeculectomy and deep sclerectomy, which have been in use for many decades. They indicate that these procedures significantly improve blood flow parameters in vessels that supply the optic nerve [4,42,43,44].

This raises the question of whether and how canaloplasty enhances the natural outflow pathway for aqueous humor (AH) and affects the extraocular circulation, with the understanding that it contributes less to lowering IOP than trabeculectomy.

The objective of this study was to assess the impact of ab externo canaloplasty on ocular blood flow parameters in patients with POAG at a three-month follow-up.

## 2. Materials and Methods

### 2.1. Study Design

A prospective, nonrandomized, single-center clinical study was conducted in the Department of Ophthalmology and the Department of Radiology of the University Clinical Hospital in Bialystok between 22 November 2022, and 1 August 2024. The study was registered on the Clinical Trials online database of the National Library of Medicine, with the registration number NCT05366647. The trial has been approved by the Bialystok Medical University Bioethics Committee (consent no. APK.002.400.2021 and APK.002.100.2024) and was carried out in accordance with the Good Clinical Practice Guidelines and the Declaration of Helsinki. Each subject enrolled in this study signed an informed consent form which detailed the nature of this study and the subject’s right to withdraw from the project at any time.

### 2.2. Study Group

The trial included 25 eyes in 25 patients with early or moderate POAG according to the Hoddap–Anderson–Parrish classification who were eligible for surgical treatment with ab externo canaloplasty, simultaneous cataract removal by phacoemulsification, and implantation of an artificial intraocular lens (IOL) (phacocanaloplasty). All surgeries were performed by the same proficient operator (JK); a detailed description of the surgical technique is provided below. The selection of this specific surgical approach was based on a comprehensive multifactorial evaluation, integrating both clinical indicators and individual patient attributes. The decision-making process was significantly influenced by the surgeon’s experience and the anatomical peculiarities of the ocular structures.

### 2.3. Inclusion Criteria

The study group was selected according to the following inclusion criteria:

- Age over 21 years;
- Diagnosis of POAG of early or moderate stage;
- Failure to normalize intraocular pressure despite maximum tolerated hypotensive therapy;
- Presence of characteristic retinal nerve fiber layer (RNFL) defects in optical coherence tomography (OCT);
- Presence of characteristic visual field defects, defined as MD (mean deviation) of ≤−12 dB, in perimetry performed with ZEISS Humphrey Field Analyzer (Carl Zeiss AG, Oberkochen, Germany) using 30-2 SITA Standard Test; with progression of the condition assessed by three perimetry measurements within 12 months;
- Informed written consent to participate in this study;
- Declaration of attendance at scheduled follow-up visits within 12 months after surgery.

### 2.4. Exclusion Criteria

The following exclusion criteria were applied:

- Visual acuity less than “counting fingers in front of the eye” (logMAR 2.0) on Bailey-Lovie Chart (Precision Vision, Woodstock, IL, USA);
- Anterior chamber angle less than a Shaffer grade 3 in all four quadrants as determined by gonioscopy;
- Advanced glaucoma;
- Other types of glaucoma besides POAG;
- History of laser therapy or previous eye surgery;
- Hemodynamically significant stenosis or obstruction of the carotid arteries;
- Lack of consent to participate in this study.

### 2.5. Examination Scheme

#### 2.5.1. Qualification for Surgery

The patients were eligible for surgical intervention up to 14 days prior to the scheduled procedure. At the time of qualification, all patients underwent a comprehensive ophthalmic examination. Furthermore, globe biometry measurements were conducted, including axial length, keratometry, and pachymetry. IOL power was calculated using the SRK/T formula. A detailed medical history was also obtained regarding the number and type of glaucoma medications, their tolerability, and the schedule of use. In addition, the medical history included information on any general illnesses, previous ophthalmic procedures, and diseases. Consistent with ethical principles and patient welfare, the implementation of a washout period for topical glaucoma drugs was excluded from the study design.

The baseline ophthalmic examination included the following:

- Assessment of best corrected visual acuity (BCVA) based on the Snellen chart;
- Intraocular pressure (IOP) measurement using Goldmann applanation tonometry (Applanation Tonometer 900, Haag-Streit Group, Köniz, Switzerland) between 8 and 10 a.m.;
- Slit-lamp examination, including evaluation of the anterior and posterior segments of the eye (assessment of the cup/disk ratio), using a Volk lens (Volk Optical Inc., Mentor, OH, USA) after pharmacological mydriasis.
- Detailed ophthalmic examination included the following:
- Gonioscopy with angle grading (Shaffer classification);
- Visual field examination with Humphrey perimeter (Carl Zeiss AG, Jena, Germany) with the SITA Standard 30-2 program. The MD index, expressed in decibels (dB), which describes the average loss of retinal sensitivity, was used to assess the severity of glaucoma. A visual field loss, defined as an MD −12 dB, was indicative of low or moderate glaucoma;
- OCT examination (Heidelberg Engineering OCT Spectralis) with RNFL evaluation;
- Color Doppler ultrasound examination of the common carotid artery (CCA), internal carotid artery (ICA), ophthalmic artery (OA), central retinal artery (CRA), and short posterior ciliary arteries (SPCAs) on both sides.

Color Doppler Imaging (CDI) was conducted consistently at the same time of day, between 9:00 and 11:00 a.m., by the same highly experienced radiologist (AU) using a TOSHIBA Aplio 400 ultrasonograph (Toshiba, Tokyo, Japan) with a 7.5 MHz linear probe. Before assessing the hemodynamic status of the ocular vessels, a color Doppler examination of the carotid arteries was performed to identify any potential alterations that could influence the ocular circulation. The principal study comprised the measurement of blood flow parameters in the OA, CRA, and SPCAs in both eyes.

The examined vessels were identified based on their position, the direction of blood flow (the arterial flow toward the probe was observed to be red, while the venous flow from the probe in the opposite direction was noted to be blue) and the relation to the globe and the optic nerve. The OA was identified as the largest arterial vessel of the orbit, as indicated by the red coloration. Upon examination, the Doppler gate was positioned at a distance of 10–25 mm behind the globe. The CRA is a small arterial vessel (red) situated in the midline of the nerve. To identify this vessel, it was beneficial to obtain a simultaneous flow spectrum from the central retinal vein (blue). Both vessels were examined at a distance of 5–10 mm behind the posterior ocular wall. SPCAs were located on the temporal and nasal sides of the optic nerve, appearing as red dots in close proximity to the nerve. Given the considerable number of ciliary arteries, the vessel exhibiting the most robust blood flow signal was subjected to analysis. The assessment of blood flow parameters in the CRA was conducted at Doppler angle values spanning the range of 0–200. This approach enabled the attainment of the maximum Doppler shift values. For OAs and SPCAs, the angle size was set at 10–450. The width of the Doppler gate/sample was fixed at 1.5 mm.

The examination was conducted in the supine position, with the head positioned at an angle of 30° relative to the body. The patients were instructed to close their eyes and maintain a fixed gaze in front of them. This was followed by the application of a specialized ultrasound gel to the eyelids. The probe was applied with minimal pressure to avoid any compression of the globe.

The analysis of the spectrum of blood flow in individual vessels included the measurement of PSV, EDV, and RI velocities. The velocities are presented in centimeters per second (cm/s). RI is a dimensionless value that ranges between 0 and 1. A value of 0 indicates no resistance in the vessel, while a value of 1 indicates a very high level of vascular resistance. PSV and EDV were measured at the peak of systole and at the end of diastole, respectively. RI was calculated using the following formula:

RI = (PSV − ESV)/PSV.

This value was indicative of vascular resistance.

A Doppler examination of the ocular vessels was conducted in accordance with the ALARA (As Low As Reasonably Achievable) principle, with the technical parameters of the apparatus maintained at a level appropriate for ocular diagnostics.

#### 2.5.2. Day of Surgery: Description of Surgical Technique

Ab externo canaloplasty was performed in eyes that had undergone uneventful, standard phacoemulsification and intraocular lens implantation under retrobulbar anesthesia with 3.5 mL 2% xylocaine. The technique of canaloplasty has been previously documented in the literature [45,46]. In brief, a corneal traction suture was placed at the 12 o’clock position to facilitate inferior rotation of the globe. Subsequently, the conjunctiva and Tenon’s capsule were incised at the limbus, and a superficial flap of the sclera measuring 5.0 by 5.0 mm and about one-third of the sclera thickness (200–250 μm) was dissected. Subsequently, a deep flap measuring approximately 4.0 by 4.0 mm was sectioned just above the choroid, thereby exposing the SC and the trabecular–Descemet window. A microcatheter equipped with a fiberscope illumination (iTrack 250A, iScience International, Menlo Park, CA, USA) was inserted into the SC and maneuvered in a gentle, circumferential manner (360°). Once the microcatheter had successfully bypassed the canal, a double-loop knot was affixed to the tip of the microcatheter with a 10/0 polypropylene suture (10/0 polypropylene thread; Prolene, Ethicon Inc., Johnson & Johnson, New Brunswick, NJ, USA). As the microcatheter traversed the SC, a viscoelastic substance was injected at two-hour intervals to facilitate expansion and maintain the canal’s patency. The suture was then tightened and knotted to gently distend the trabecular meshwork inwards. At the end of the procedure, the superficial flap was repositioned and tightly sutured with 5 interrupted 10/0 monofilament nylon sutures. The conjunctiva was sutured using absorbable sutures over the limbus.

Patients were prescribed topical drops with an antibiotic (2 weeks), a steroid (4 weeks), and a non-steroidal anti-inflammatory drug (4 weeks) after the surgery. Previous topical glaucoma treatment was discontinued on the day of surgery.

#### 2.5.3. Postoperative Schedule

During the postoperative period, follow-up visits were conducted on days 1, 7, and 14, as well as 1 and 3 months after surgery. During the first three visits, the patients underwent a basic ophthalmic evaluation. During subsequent visits, which occurred one and three months after surgery, basic examinations were accompanied by a detailed ophthalmic evaluation.

The primary variables studied were a reduction in IOP and a reduction in the number of glaucoma drops administered postoperatively. Total surgical success was defined as IOP < 18 mmHg or a 20% reduction in IOP compared to baseline. Satisfactory surgical success: the same IOP parameters with the administration of up to two types of glaucoma drops. Improvement in BCVA and the stability of MD and RNFL parameters were defined as treatment safety outcomes.

The efficacy of surgical intervention was also assessed at an early stage based on the observed improvement in the PSV and EDV parameters, as well as a reduction in RI in the OA, CRA, and SPCA assessed by CDI in the operated eye.

### 2.6. Statistical Analysis

Statistical analysis was performed with R software, version 4.1.2. Nominal variables were expressed as *n* (%); numeric variables were presented as mean and standard deviation or median and interquartile range, depending on distribution. Distribution normality was verified with Shapiro–Wilk test; skewness and kurtosis were used for additional confirmation. Comparisons were performed with tests for dependent samples (paired *t*-test or Wilcoxon test). Correlation between two numeric variables was analyzed with the Spearman method due to non-normal distributions of some variables. All tests were assumed to reach significance when *p* < 0.05. The incidence of surgical success was calculated with the Kaplan–Meier survival analysis method, including 95% confidence intervals.

## 3. Results

### 3.1. Characteristics of the Group

A total of 25 subjects, comprising 17 women and 8 men, aged 45 to 83 (mean age 67.0 ± 10.54 years), were included in this study. Of these, eighteen (72%) had hypertension, four (16%) had diabetes, and nine (36%) had lipid disorders. Additionally, five (20%) of the subjects were smokers.

### 3.2. Change in Selected Parameters in Operated Eyes from Before Surgery (Baseline) to Each Measured Time Point

The observation period was three months. Selected parameters, indicating either directly or indirectly the stage of glaucoma (IOP, BCVA, MD, RNFL), were compared between the baseline and those obtained one and three months after the surgery (Table 1).

A reduction in IOP and an improvement in mean deviation (MD) were observed at one month following canaloplasty. However, these changes were not statistically significant until the control visit at three months post-surgery (*p* = 0.049 and *p* = 0.006, respectively). Significant increases in BCVA and RNFL were observed at each postoperative control visit compared to baseline (*p* < 0.001 and *p* = 0.001 at 1 month and *p* = 0.002 and *p* = 0.002 at 3 months, respectively).

The mean number of topical glaucoma medications used prior to surgery was 1.84: 22 patients (88%) used topical prostaglandin (one time daily), 8 patients (32%) used a topical αlpha-2 agonist (two times daily), 8 patients used a topical beta-blocker (two times daily), and 8 patients (32%) used topical dorzolamide (two times daily). Among the 24 patients (96%) who discontinued the use of glaucoma drops after surgery, a statistically significant decrease in the number of medications was observed (MD = −1.67, CI95 = [−2.11; −1.22], *p* < 0.001).

**Figure 1:**
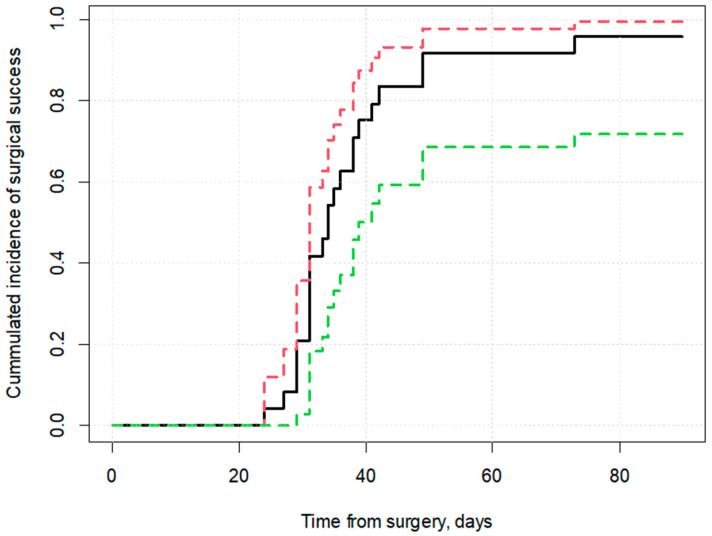
Kaplan–Meier curve for incidence of surgical success. Notes: Surgical success was defined as IOP < 18 mmHg or a 20% reduction in IOP compared to baseline. Continuous line represents the proportion of patients who achieved surgical success. Dashed lines represent 95% confidence interval.

Median time to surgical success, based on the Kaplan–Meier model, was 34 days (CI 95%: 31–39) (Figure 1).

### 3.3. Change in PSV, EDV, and RI in OA, CRA, and SPCAs in Operated Eyes from Before Surgery (Baseline) to Each Measured Time Point

Prior to surgical intervention, no statistically significant differences were observed in the blood flow parameters of the OA, CRA, and SPCAs between the operated eye and the contralateral, unoperated eye of the same patient.

No significant changes were observed for PSV, EDV, and RI in the operated eye between baseline and the control visit 1 and 3 months after surgery (Table 2, Table 3 and Table 4).

### 3.4. Correlation Between Glaucoma Parameters and Flow Parameters Before Surgery

**Figure 2:**
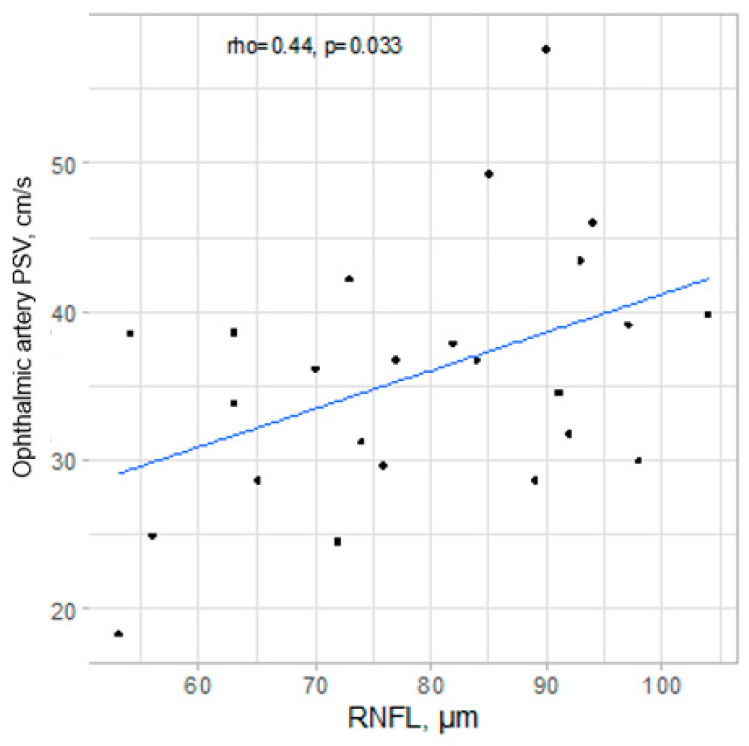
Scatterplot presenting relationship between RNFL and PSV in ophthalmic artery in eyes before surgery.

A positive correlation of moderate strength between RNFL and ophthalmic artery PSV was confirmed for operated eyes, rho = 0.44, *p* = 0.033. The relationship is presented visually in Figure 2.

### 3.5. Comparison of Intraocular Pressure and Flow Parameters Between Operated and Unoperated Eyes Before Surgery

Statistically significant differences were observed between the PSV in the SPCAs and the preoperative IOP values of the eye that was qualified for operation and the contralateral eye. IOP in the operated eyes was found to be significantly higher in comparison to unoperated eyes (MD = 1.94, CI95 [1.01; 2.86], *p* < 0.001). Similarly, the short posterior ciliary artery PSV for operated eyes was observed to be significantly higher in comparison to unoperated eyes (MD = 1.35, CI95 [0.09; 2.61], *p* = 0.037).

No other statistically significant correlations were identified between the examined parameters, either during the preoperative period or in the postoperative follow-up examinations.

## 4. Discussion

The objective of this study was to determine whether a nonpenetrating procedure acting through a physiological mechanism—ab externo canaloplasty—affects ocular blood flow parameters in vessels involved in optic nerve vascularization. A three-month follow-up revealed no statistically significant differences between parameters PSV, EDV, and RI in OA, CRA, and SPCAs before and after surgical treatment. Furthermore, no significant correlations were identified between CDI parameters of the ocular circulation and factors directly and indirectly describing glaucoma severity, including BCVA, IOP, RNFL, and MD.

Ocular blood flow is dependent on ocular perfusion pressure (OPP), which is defined as two-thirds of the difference between mean arterial pressure (MAP) and IOP. Consequently, a reduction in IOP should result in an increase in OPP and, therefore, in optic nerve blood flow. The existing literature suggests that a reduction in OPP, which is associated with a constant decrease or diurnal variation in blood pressure, may be a predisposing factor to the progression of glaucomatous neuropathy [47,48,49]. In a meta-analysis conducted by Kim et al. [50], a reduced OPP was observed in patients with POAG, associated with higher IOP in comparison to the healthy population. In this group of patients who did not develop advanced glaucomatous neuropathy, the identification of vascular risk factors (in particular, inadequately treated hypertension) and the evaluation of ocular perfusion parameters in CDI could become valuable diagnostic factors in the process of qualification for antiglaucoma surgery to select the most optimal surgical technique. In our study, the mean reduction in IOP three months after ab externo canaloplasty was no greater than 2–3 mmHg compared to the baseline measurement, with almost 100% reduction in medication burden, indicating that this procedure is suitable for patients with a lower baseline IOP, intolerant to pharmacological treatment, in whom surgical success can be achieved even with a reduction in low or moderate IOP.

The impact of SC procedures on ocular blood flow measured by CDI has not yet been investigated. To the best of our knowledge, our study is the first to address this issue. The only available studies describe the effects of trabeculectomy and deep sclerectomy on ocular perfusion. Both procedures appear to have a beneficial effect on blood flow in the vessels that supply the optic nerve [4,42,43,44]. It is plausible that this is due to the fact that these procedures have a more pronounced impact on lowering IOP than nonpenetrating glaucoma surgery.

Canaloplasty is a surgical procedure designed to improve aqueous outflow by inserting a microcatheter into the circumference of the SC, delivering a viscoelastic material, and subsequently suturing to apply tension to the inner wall of the SC. This results in distension of the trabecular meshwork and increased outflow through collector channels of the conventional pathway [51]. The SC is located at the drainage site of the iridocorneal angle. This particular region is circumscribed by the trabecular meshwork (TM), located above the SC. Together, they form the standard outflow pathway, which is responsible for excreting between 50% and 90% of the AH [52].

The SC is characterized by the presence of a continuous layer of endothelium with tight junctions, which is organized into an outer and an inner wall. The endothelium in direct contact with the juxtacanalicular tissue is defined as the inner wall and it plays a fundamental role in the specialized function of the SC. The outer wall is constituted by the remaining endothelial cells. The endothelial cells of the inner wall are subjected to a distinct biomechanical microenvironment due to their proximity to the juxtacanalicular tissue, which is characterized by a basal–apical pressure gradient. This distinctive environment is responsible for the formation of pores and giant vacuoles within the endothelial cells of the inner wall. Giant vacuoles are not intracellular structures; rather, they represent interstitial spaces between the inner wall cells and the extracellular matrix. These vacuoles are dynamic structures that demonstrate an increase in both number and size in response to elevated IOP. Furthermore, the pores, which are perforations in the inner wall with a diameter ranging from 0.6 to 3 μm, facilitate the transport of AH from the giant vacuoles into the SC. They serve as one-way valves, preventing backflow. In areas where higher filtration is required, SC cells undergo greater deformation due to the formation of giant vacuoles. This increased strain promotes the development of pores, thereby enhancing the filtration pathway for AH. In the course of glaucoma, the functionality of this intricate system is compromised, leading to an increase in IOP [52,53,54,55]. On the contrary, the unconventional “uveoscleral” route facilitates the exit of AH from the eye through the interstitial areas of the ciliary body, functioning independently of IOP [56]. Canaloplasty leaves the outer wall of the SC and the structures of the distal outflow tract of the AH, so distal resistance still remains, resulting in a higher IOP than if these structures were bypassed directly into the subconjunctival space, as in bleb procedures. This type of resistance can even reach 40% of the total resistance. Outflow through the SC is less important in deep sclerectomy and trabeculectomy, but crucial in canaloplasty and other SC-based procedures.

From an embryological and morphological perspective, the SC endothelium exhibits characteristics that are shared with both vascular and lymphatic endothelium. This distinctive phenomenon presents a challenge in classifying this type of tissue, which in turn limits our understanding of its physiology [56,57]. The available evidence indicates that glaucoma is associated with damage and remodeling of the SC endothelium. These processes, including fibrosis, increased stiffness, and a reduction in the number of giant vacuoles and pores, are directly related to impaired aqueous outflow from the eye [53,58,59]. In the aforementioned publications, the authors observed analogous abnormalities in ocular blood flow and vascular endothelial dysfunction in glaucoma patients with circulatory risk factors. Canaloplasty, whose primary objective is to dilate the SC, contributes to the improvement of AH outflow and the reduction in IOP; however, it does not restore remodeled endothelial cells. It can be deduced that in patients with extensive endothelial cell damage and dysfunction, resulting in subsequent SC insufficiency and advanced glaucomatous neuropathy, the efficacy of canaloplasty is significantly limited. In this group of patients, similar to patients with significant disturbances in ocular blood flow, it would be beneficial to consider the potential use of more invasive, penetrating techniques.

It is important to note that canaloplasty is a technique that is primarily indicated for patients with early to moderate glaucoma. Consequently, the expected hypotensive effect is lower, comparing to trabeculectomy, which is the preferred surgical option for patients with advanced glaucoma neuropathy [60,61,62]. The results of this study confirm the efficacy of phacocanaloplasty in reducing IOP in patients with POAG. This is evidenced by a statistically significant decrease in IOP three months after surgery. Furthermore, other parameters, such as MD and RNFL thickness, improved significantly after surgery. The number of topical glaucoma drops was also significantly reduced, with 96% of operated patients not requiring glaucoma treatment three months after the phacocanaloplasty procedure. The hypotensive effect of both canaloplasty and phacocanaloplasty has been extensively documented in the literature. Authors of retrospective and prospective studies emphasize the efficacy of this surgical approach in long-term inhibition of the progression of glaucomatous neuropathy by effectively lowering IOP levels [63,64,65,66,67,68]. It is also noteworthy that this procedure is distinguished by a favorable safety profile and a relatively low complication rate in comparison to other, more invasive glaucoma procedures [51,69,70].

Other authors also searched for the physiological mechanism in glaucomatous eyes. The globe, as an individual organ, is subject to a circadian rhythm. Diurnal changes in the shape and volume of its tissues, caused by fluctuations in IOP and dynamic changes in the cardiovascular system, are referred to as ocular pulse (OP) [71]. Mansouri et al. [71] studied OP with the Sensimed Triggerfish contact lens sensor (STCLS) that enables the 24 h measurement of this parameter’s amplitude through the detailed analysis of changes in corneal shape, facilitating the daily analysis of IOP and ocular dynamics correlated with cardiovascular rhythm. This may prove particularly useful in patients who, despite exhibiting normal IOP measurements during ophthalmology visits, develop progression of glaucomatous neuropathy [72]. Other authors have emphasized the potential utility of ocular pulse measurement in the diagnosis and monitoring of glaucomatous neuropathy, taking into account the complex etiology of the condition [73,74,75]. In another study, Rękas et al. [76] examined OP amplitude correlated with electrical heart activity in Holter electrocardiography with the aim of evaluating the surgical effect of glaucoma patients after canaloplasty and phacocanaloplasty. The increased ocular pulse observed in glaucoma patients may be the result of several factors, including impaired outflow of aqueous fluid through natural pathways, increases in IOP, and abnormalities in choroidal blood flow, as the authors themselves noted. The results of the study demonstrated a statistically significant reduction in IOP fluctuations at both 3 and 12 months post-surgery compared to the baseline measurement. This was observed in a 24 h measurement of corneoscleral limbus area straining using the Sensimed Triggerfish contact lens sensor (Sensimed, Lausanne, Switzerland). Nevertheless, despite a statistically significant reduction in IOP and operational success, signal variances were not entirely eliminated. As the authors hypothesize, a more invasive, perforating technique such as trabeculectomy could be utilized to achieve this state. It is plausible that the OP fluctuations observed in patients in the postoperative period were due to blood flow disturbances in the choroidal circulation. Based on the findings of the study, it can be hypothesized that canaloplasty was insufficiently effective to affect these fluctuations. This illustrates an analogy with our findings, in which the canaloplasty procedure was found to be ineffective in improving flow parameters in the ocular vessels.

In examining the circulatory status of ocular vessels, it is essential to consider the microcirculation of the retina, choroid, and optic nerve. These structures represent the final segment of the ocular arterial circulation, receiving blood supply from the CRA and SPCAs [77], the parameters of which were analyzed in our study. The phenomenon of microvasculature dropout, observed in patients with glaucoma, has been extensively described in the literature [78,79,80,81,82,83,84,85]. The authors devote particular attention to the microvasculature disruption observed in patients with preperimetric glaucoma and the correlation of microvasculature dropout with the severity of the condition. This phenomenon may be of value in a diagnostic and prognostic context. The effect of various surgical procedures on microvasculature and vessel density has been documented in the literature. However, the results of these studies are often contradictory [78,86,87,88,89,90,91,92,93]. Nevertheless, trials conducted on an analogous model for the canaloplasty procedure are not yet available. The majority of publications related to glaucoma and its vascular factors tend to focus on a single section of the ocular circulation. A comprehensive analysis of ocular vascular blood flow parameters, including the entire vasculature—from the ophthalmic artery to the microcirculation of the choroid, retina, and optic nerve—could provide valuable information on the primary circulatory changes in the course of glaucoma, particularly in patients with the preperimetric type of the disease. This would significantly improve our understanding of the vascular risk factors for glaucoma.

## 5. Limitations

It should be noted that our publication is subject to certain limitations, which we are aware of. The results of this study should be regarded as preliminary. Given the relatively small size of the study group and the short follow-up period, we have decided to present the preliminary results of our study. The absence of analogous research models in the existing literature precludes the possibility of relating the data from our analysis to other studies. This further motivated us to publish the preliminary results. Currently, we are in the process of expanding the study group and extending the follow-up period to 12 months. Additionally, we intend to analyze other nonpenetrating surgical techniques.

Another limitation is the lack of available standards and norms for CDI blood flow parameters in the ocular arteries, due to their highly individual nature. Consequently, an additional analysis of blood flow parameters in the contralateral eye in the same patient was conducted, and the results demonstrated no significant differences between eyes.

## 6. Conclusions

POAG treatment lasts a patient’s lifetime and therefore requires a holistic approach with consideration of their general health, as well as the severity of the glaucoma. Given the considerably less invasive nature of canaloplasty in comparison to trabeculectomy, along with the reduced hypotensive effect and the lack of evidence that it improves blood flow in the vessels supplying the optic nerve, it is essential that patients are carefully qualified for this type of procedure. In patients with advanced glaucoma and concomitant vascular risk factors, the surgical option with the most effective hypotensive outcome, such as trabeculectomy, should be considered. A more aggressive surgical approach may also be indicated for a specific group of patients with underlying cardiovascular diseases, such as hypertension, or who are predisposed to fluctuating blood pressure. These patients may not have developed advanced glaucomatous neuropathy despite elevated IOP; however, they may be at risk of progression of optic nerve damage if treated inadequately. Consequently, it is essential to subject patients to rigorous qualification for a specific surgical procedure, with particular attention paid to a comprehensive medical history. Further, larger, multi-center studies are necessary to analyze this issue in greater detail.

## Figures and Tables

**Table 1:** Evolution of IOP, BCVA, RNFL, and MD in operated eye with time.

Variable	Baseline	1 Month			3 Months		
		Mean ± SD/Median (IQR)	MD (95% CI)	*p*	Mean ± SD/Median (IQR)	MD (95% CI)	*p*
IOP ^1,^* (mmHg)	17.42 ± 4.34	15.54 ± 3.79	−1.84 (−3.81; 0.13)	0.066	15.81 ± 3.58	−2.00 (−3.99; −0.01)	**0.049**
BCVA ^2,^**	0.90 (0.70; 0.90)	1.00 (1.00; 1.00)	0.10 (0.15; 0.30)	**<0.001**	1.00 (1.00; 1.00)	0.10 (0.10; 0.30)	**0.002**
RNFL ^2,^** (μm)	70.50 (57.75; 77.75)	78.00 (65.00; 88.00)	7.50 (2.50; 9.50)	**0.001**	76.00 (60.50; 87.00)	5.50 (1.50; 7.00)	**0.002**
MD ^2,^** (dB)	−6.16 (−8.48;−2.91)	−4.82 (−7.74;−2.40)	1.34 (−0.27; 1.58)	0.188	−3.36 (−6.51;−1.77)	2.80 (0.33; 2.40)	**0.006**

Notes: * mean + SD; ** median (IQR); ^1^ SD—standard deviation; MD—mean change vs. baseline; CI—confidence interval. Comparisons made with paired *t*-test; ^2^ IQR—interquartile range; MD—median change vs. baseline; CI—confidence interval. Comparisons made with Wilcoxon test.

**Table 2:** Evolution of PSV in operated eye with time.

PSV, cm/s	Mean ± SD/Median (IQR)	MD (95% CI)	*p*
Ophthalmic artery			
Baseline	34.15 (30.37; 39.10)		
1 month	31.60 (28.90; 35.25)	−2.55 (−7.20; 1.30)	0.160
3 months	32.90 (28.07; 37.08)	−1.25 (−6.20; 5.10)	0.508
Central retinal artery			
Baseline	9.18 ± 2.11		
1 month	8.68 ± 3.22	−0.40 (−1.53; 0.72)	0.464
3 months	9.42 ± 2.84	0.20 (−0.96; 1.36)	0.722
Short posterior ciliary artery			
Baseline	13.36 ± 2.98		
1 month	13.10 ± 2.74	0.00 (−1.23; 1.23)	0.994
3 months	12.28 ± 2.97	−0.25 (−1.69; 1.19)	0.722

Notes: SD—standard deviation; IQR—interquartile range; MD—mean or median change vs. baseline; CI—confidence interval. Comparisons made with paired *t*-test or Wilcoxon test.

**Table 3:** Evolution of EDV in operated eye with time.

EDV, cm/s	Mean ± SD/Median (IQR)	MD (95% CI)	*p*
Ophthalmic artery			
Baseline	7.75 (5.70; 9.15)		
1 month	7.40 (6.20; 8.55)	−0.35 (−1.65; 1.00)	0.573
3 months	7.70 (5.53; 9.00)	−0.05 (−1.90; 1.90)	0.603
Central retinal artery			
Baseline	2.50 (2.20; 2.80)		
1 month	2.25 (1.87; 2.95)	−0.25 (−0.45; 0.30)	0.537
3 months	2.70 (2.15; 3.45)	0.20 (−0.10; 0.75)	0.133
Short posterior ciliary artery			
Baseline	4.00 ± 1.32		
1 month	3.90 ± 1.36	−0.03 (−0.65; 0.60)	0.934
3 months	3.96 ± 1.51	−0.03 (−0.66; 0.60)	0.931

Notes: SD—standard deviation; IQR—interquartile range; MD—mean or median change vs. baseline; CI—confidence interval. Comparisons made with paired *t*-test or Wilcoxon test.

**Table 4:** Evolution of RI in operated eye with time.

RI, cm/s	Mean ± SD/Median (IQR)	MD (95% CI)	*p*
Ophthalmic artery			
Baseline	0.79 (0.75; 0.81)		
1 month	0.78 (0.74; 0.80)	−0.01 (−0.03; 0.01)	0.117
3 months	0.78 (0.73; 0.83)	−0.01 (−0.03; 0.03)	0.513
Central retinal artery			
Baseline	0.72 (0.66; 0.76)		
1 month	0.72 (0.66; 0.74)	0.00 (−0.04; 0.02)	0.300
3 months	0.67 (0.62; 0.72)	−0.05 (−0.07; 0.01)	0.191
Short posterior ciliary artery			
Baseline	0.70 ± 0.08		
1 month	0.70 ± 0.07	0.01 (−0.02; 0.04)	0.583
3 months	0.68 ± 0.07	0.00 (−0.03; 0.03)	0.948

Notes: SD—standard deviation; IQR—interquartile range; MD—mean or median change vs. baseline; CI—confidence interval. Comparisons made with paired *t*-test or Wilcoxon test.

## Data Availability

All materials and information will be available upon e-mail request from the corresponding author.
